# Assessing Bacterial Viability and Label Accuracy in Human and Poultry Probiotics Sold in the United Kingdom

**DOI:** 10.3390/microorganisms13081933

**Published:** 2025-08-19

**Authors:** Mostafa Waleed Taha, Danielle J. C. Fenwick, Emma C. L. Marrs, Abdul Shakoor Chaudhry

**Affiliations:** 1 School of Natural and Environmental Sciences, Newcastle University, Newcastle upon Tyne NE1 7RU, UK; m.w.t.taha2@newcastle.ac.uk; 2 Microbiology Research Department, Freeman Hospital, Newcastle upon Tyne NE7 7DN, UK; danielle.fenwick@nhs.net (D.J.C.F.); e.marrs@nhs.net (E.C.L.M.)

**Keywords:** probiotics, label accuracy, quality control, agar media, *Bacillus* spp. spores, viable bacterial count, MALDI-TOF MS

## Abstract

Accurate label claims are essential for consumer trust in probiotic efficacy, yet limited datasets are available for poultry formulations marketed in the United Kingdom. We quantified and identified the viable bacteria in twelve commercial probiotics, seven for poultry and five for human use, using selective plate counts and MALDI-TOF MS. Observed colony forming units (CFU) were compared with declared values using one-sample *t*-tests, adopting a practical acceptance range of ±0.5 log CFU. Poultry products largely met or exceeded their labels (e.g., P5: 1.4 × 10^10^ CFU g^−1^ vs. 2 × 10^9^ CFU g^−1^ declared), whereas human products delivered greater variability in both species composition and stated CFU count; one contained no detectable viable bacteria. All products deviated significantly from their label claims (*p* < 0.05); however, 11 of 12 met the ±0.5 log_10_ CFU benchmark—10 within the range and 1 above its “≥” value—leaving only one probiotic below the threshold. MALDI-TOF MS confirmed the presence of most labelled species, though *Bifidobacterium bifidum* was absent from one human product and *Bacillus* isolates were re-assigned to *B. velezensis*/*B. amyloliquefaciens*. These findings indicate robust quality assurance in UK poultry probiotics, but substantial under-delivery in the human probiotics, underscoring the need for harmonized viability standards and tighter post-market surveillance.

## 1. Introduction

According to the FAO/WHO, probiotics are defined as “live microorganisms which, when consumed in adequate amounts, confer health benefits on the host” [1]. The presence of viable microorganisms is therefore crucial [2]. Beneficial bacteria must remain alive in commercial products to deliver effects such as improving gut health, stimulating immunity, competing with pathogens and producing bioactive metabolites [2,3].

Label claims for viable counts and species, however, are often unreliable because most probiotic products are regulated as foods rather than medicines [4]. Numerous surveys of human probiotics have documented large discrepancies between declared and observed viable counts, with some products lacking the stated species altogether [2,5,6,7,8,9,10]. By contrast, data on poultry probiotics, particularly those marketed in the United Kingdom, remain scarce. The only UK study identified evaluated seven human products in 2016; fewer than half of those met their declared culture concentrations [11]. No recent, systematic assessment of poultry formulations has been published yet.

Enumeration of probiotic organisms is typically culture-dependent, employing selective agar media to determine colony-forming units (CFU) per dose [2,7,8,9,12,13]. Plate counting is the sole International Organization for Standardization (ISO)-validated standard for probiotic enumeration [14] and is endorsed by the European Commission for use in animal-feed research [15]. For species confirmation, matrix-assisted laser desorption/ionisation time-of-flight mass spectrometry (MALDI-TOF MS) offers rapid, reliable and cost-effective identification and is now routinely applied in diagnostic microbiology [16,17,18].

This study therefore evaluated the label accuracy of twelve UK-market probiotics—seven formulated for poultry and five for humans—using plate enumeration on selective agars and MALDI-TOF MS. We hypothesized that (i) observed viable counts would be significantly different than declared values and (ii) some labelled species would be absent. The results provide a much-needed baseline for functional studies and for post-market quality control of commercial probiotics before their dietary use.

## 2. Materials and Methods

### 2.1. Ethics Statement

Ethical approval was obtained from the Animal Welfare and Ethical Review Body at Newcastle University, United Kingdom (Project ID #948).

### 2.2. Probiotic Products Selection and Handling

Twelve commercial probiotic products (coded P1–P12; seven intended for poultry and five for human use) were obtained in the United Kingdom via commonly used online sources available to UK consumers for both human and poultry probiotics. Selection was by convenience sampling, stratified to cover both applications (human and poultry), multiple formulation types (capsule, tablet/chewable, powder) and differing taxa/technologies (non-spore-forming lactic acid bacteria and spore-forming Bacillus; single- and multi-strain).

For each product, one commercial batch was analysed. From that batch, technical replicates were prepared in parallel (*n* = 3 for multi-strain products; *n* = 4 for single-strain products). Lot numbers and expiry dates were recorded prior to analysis. All products were factory-sealed on arrival and tested before expiry. To ensure the accurate growth and identification of the target organisms, specific selective media and incubation conditions were used, as detailed in Table 1. Declared total counts and labelled species are listed in Table 2 (poultry probiotics) and Table 3 (human probiotics). Storage metadata (expiry date, label storage instruction, actual storage temperature and storage time prior to testing) are provided in Supplementary Table S1 and cross-referenced to product codes and their intended use (human or poultry) in Table 4.

Products were stored in their original packaging and under the manufacturer’s recommended storage conditions.

### 2.3. Optimisation of Selective Media and Incubation Conditions

Agar media were prepared according to manufacturer’s instructions. Molten agar (~55 °C) was poured into sterile 90 mm three-vent Petri dishes (Thermo Scientific, Newport, UK, P11309283) within a Class II microbiological safety cabinet (MSC 12, Jouan SA, Saint-Herblain, France). Selective supplements (e.g., vancomycin, lithium-mupirocin) were applied as detailed in Table 1, which also lists the target organisms and their incubation conditions.

A short pilot study was run for every probiotic to assess growth and selectivity on the chosen selective agars and to establish their appropriate dilution range. For products P9 and P12 (containing *Lactobacillus salivarius*, *Bifidobacterium animalis* and *Enterococcus faecium*), standard Bifidobacterium Selective Medium (BSM) agar plus BSM supplement supported *B. animalis* but also permitted *E. faecium* growth; selectivity was restored by adding lithium-mupirocin (5 mL/100 mL), as previously reported [19]. Conversely, supplementing MRS (De Man–Rogosa–Sharpe) agar with vancomycin (20 mg L^−1^) inhibited *E. faecium* while allowing *L. salivarius* to grow [20].

For *Bacillus* spp. products, Tryptone Soya Agar (TSA) plates were pre-incubated at 37 °C for approximately 13 h, then inoculated and cultured at 33 °C instead of 37 °C. This two-step protocol prevented *B. subtilis* swarming and enabled accurate enumeration; swarming was not observed under the adjusted conditions.

**Table 1:** Agar media and incubation conditions for the assessed probiotic bacteria.

Agar Medium	Supplements	Target Organism(s)	Incubation Conditions
BSM Agar (Sigma, Gillingham, UK)	BSM Supplement (Sigma, UK)	*Bifidobacterium* spp.	Anaerobic, 37 °C, 72 h
ChromoSelect Agar Base (Sigma, Gillingham, UK)	*E. faecium* selective supplement (Sigma, UK)	*Enterococcus faecium*	Aerobic, 37 °C, 24 h
MRS Agar (Sigma, Gillingham, UK)	—	*Lactobacillus* spp.	Anaerobic, 37 °C, 72 h
Tryptone Soya Agar (TSA; Thermo Scientific, Basingstoke, UK)	—	*Bacillus* spp.	Aerobic, 33 °C, 17 h ^1^
MRS Agar (Sigma, Gillingham, UK)	Vancomycin hydrochloride (20 mg L^−1^) ^2^	*Ligilactobacillus salivarius*	Anaerobic, 37 °C, 72 h
BSM Agar (Sigma, Gillingham, UK)	BSM Supplement; lithium mupirocin (5 mL/100 mL) ^3^	*Bifidobacterium animalis*	Anaerobic, 37 °C, 72 h

^1^ TSA plates were pre-incubated at 37 °C for ~13 h before use to prevent *B. subtilis* swarming, then incubated at 33 °C. ^2^ Used to selectively grow *Lactobacillus salivarius* from probiotics 9 and 12. ^3^ Used to selectively grow *Bifidobacterium animalis* only from P9 and P12.

**Table 2:** Details and viable counts of commercial poultry probiotic products.

Product Code	Species Declared on the Label	Declared Label Total (CFU/g)	MALDI-TOF MS ID ^2^	Observed Viable Plate Counts (Mean ± SD) ^3^
P3	*Bacillus subtilis*	2.5 × 10^7^	*Bacillus* spp.	1.375 × 10^7^ ± 2.06 × 10^6^
P4	*Bacillus subtilis*	3 × 10^8^	*Bacillus* spp.	1.900 × 10^8^ ± 1.82 × 10^7^
P5	*Bacillus subtilis*	2 × 10^9^	*Bacillus* spp.	1.400 × 10^10^ ± 2.58 × 10^9^
P9	*Bifidobacterium animalis*	Ratio ^1^ 3/10	*Bifidobacterium animalis*	1.300 × 10^8^ ± 2.58 × 10^7^
	*Lactobacillus salivarius*	Ratio 1/10	*Ligilactobacillus salivarius*	3.200 × 10^7^ ± 3.65 × 10^6^
	*Enterococcus faecium*	Ratio 6/10	*Enterococcus faecium*	1.055 × 10^9^ ± 4.43 × 10^7^
	Total bacteria	1 × 10^9^	Total bacteria	1.212 × 10^9^ ± 4.47 × 10^7^
P10	*Enterococcus faecium*	2 × 10^10^	*Enterococcus faecium*	5.800 × 10^10^ ± 4.54 × 10^9^
P11	*Bacillus subtilis*	2 × 10^8^	*Bacillus* spp.	5.950 × 10^8^ ± 3.10 × 10^7^
P12	*Bifidobacterium animalis*	Ratio ^1^ 3/10	*Bifidobacterium animalis*	1.080 × 10^7^ ± 1.48 × 10^6^
	*Lactobacillus salivarius*	Ratio 1/10	*Ligilactobacillus salivarius*	1.375 × 10^6^ ± 2.21 × 10^5^
	*Enterococcus faecium*	Ratio 6/10	*Enterococcus faecium*	5.300 × 10^8^ ± 4.32 × 10^7^
	Total bacteria	2 × 10^8^	Total bacteria	5.418 × 10^8^ ± 4.22 × 10^7^

^1^ For products P9 and P12, the manufacturer declared the total CFU per gram of product but did not specify individual CFU counts for each bacterial species. Instead, the label listed each species as a ratio of the total bacterial population. ^2^ Identification of isolates by matrix-assisted laser desorption/ionisation time-of-flight mass spectrometry. ^3^ Mean ± standard deviation per gram. Observed means are based on *n* = 3 replicates for multi-species products and *n* = 4 for single-species products.

**Table 3:** Details and viable counts of commercial human probiotic products.

Product Code	Form	Species Declared on the Label	Declared Label Total (CFU/Form ^1^)	MALDI-TOF MS ID ^3^	Observed Viable Plate Counts (Mean ± SD) ^4^
P1	Tablets	*Lactobacillus acidophilus*	2 × 10^11^	NA	No CFU detected ^2^
P2	Capsule	*Lactobacillus acidophilus*	ND ^5^	*Lactobacillus acidophilus*	3.483 × 10^9^ ± 2.76 × 10^8^
		*Bifidobacterium animalis*	ND	*Bifidobacterium animalis*	6.533 × 10^8^ ± 3.51 × 10^7^
		*Bifidobacterium bifidum*	ND		
		Total bacteria	1 × 10^10^	Total bacteria	4.137 × 10^9^ ± 2.96 × 10^8^
P6	Capsules	*Lactobacillus acidophilus*	ND	*Lactobacillus acidophilus*	3.300 × 10^9^ ± 1.00 × 10^8^
		*Lactobacillus salivarius*	ND	*Lactobacillus salivarius*	2.067 × 10^6^ ± 4.61 × 10^5^
		*Bifidobacterium animalis*	ND	*Bifidobacterium animalis*	2.100 × 10^7^ ± 2.00 × 10^6^
		*Lactobacillus Bulgaricus*	ND		
		Total bacteria	3 × 10^9^	Total bacteria	3.320 × 10^9^ ± 1.01 × 10^8^
P7	Capsules	*Lactobacillus acidophilus*	ND	*Lactobacillus acidophilus*	5.667 × 10^9^ ± 3.05 × 10^8^
		*Bifidobacterium bifidum*	ND	*Bifidobacterium bifidum*	8.100 × 10^8^ ± 4.35 × 10^7^
		Total bacteria	1 × 10^10^	Total bacteria	6.477 × 10^9^ ± 3.36 × 10^8^
P8	Chewable Tablets	*Lactobacillus acidophilus*	ND	*Lactobacillus acidophilus*	5.533 × 10^8^ ± 1.52 × 10^7^
		*Bifidobacterium animalis*	ND	*Bifidobacterium animalis*	2.800 × 10^7^ ± 1.00 × 10^6^
		Total bacteria	1 × 10^9^	Total bacteria	5.813 × 10^8^ ± 1.56 × 10^7^

^1^ Per form (capsule or tablet, as specified in the “Form” column, except for P1, where label showed counts per gram). ^2^ For product P1, viability testing was conducted using two sample types: (i) a single tablet, and (ii) a composite of seven tablets equivalent to 1 g, as the label declared viable count per gram. No colony growth was observed in either case on any agar medium. ^3^ Identification of isolates by matrix-assisted laser desorption/ionization time-of-flight mass spectrometry. ^4^ Mean ± standard deviation per form. Observed means are based on *n* = 3 replicates for multi-species products and *n* = 4 for single-species products. ^5^ ND = Not declared on label; the manufacturer provided a total count but did not specify counts for individual species.

**Table 4:** Observed vs. declared viable counts (log_10_ CFU) in probiotic products (P1–P12).

Product Code	Application	Declared Total Bacteria (log_10_ CFU/unit ^1^)	Observed Total Viable Plate Counts (log_10_ CFU ± SD) ^2^	*p*-Value ^3^	95% Confidence Interval (CI)	Acceptable Log_10_ Range (±0.5) ^4^	Relative Difference (%) ^5^
P1	Human	11.30	No CFU	0.000	(0.00, 0.00)	10.80–11.80	−100.00
P2	Human	10	9.61 ± 0.031	0.002	(9.53, 9.69)	9.50–10.50	−3.90
P3	Poultry	7.39	7.13 ± 0.067	0.005	(7.02, 7.24)	6.89–7.89	−3.52
P4	Poultry	8.47	8.27 ± 0.042	0.003	(8.21, 8.34)	7.97–8.97	−2.36
P5 ^6^	Poultry	min. 9.30	10.14 ± 0.081	0.001	(10.01, 10.26)	8.80–9.80	9.03
P6	Human	9.47	9.52 ± 0.007	0.023	(9.48, 9.55)	8.97–9.97	0.53
P7	Human	10	9.81 ± 0.022	0.005	(9.75, 9.86)	9.50–10.50	−1.90
P8	Human	9	8.76 ± 0.011	0.001	(8.73, 8.79)	8.50–9.50	−2.67
P9	Poultry	9	9.08 ± 0.015	0.002	(9.05, 9.10)	8.50–9.50	0.89
P10	Poultry	10.30	10.76 ± 0.033	0.001	(10.70, 10.81)	9.80–10.80	4.46
P11	Poultry	min. 8.30	8.77 ± 0.023	0.001	(8.73, 8.81)	7.80–8.80	5.66
P12	Poultry	8.30	8.73 ± 0.034	0.001	(8.67, 8.78)	7.80–8.80	5.18

^1^ As declared on label (per capsule or tablet for human probiotics, except for P1, their label showed counts per gram, therefore both units—gram and tablets—were tested for growth. Per gram for all poultry probiotics). ^2^ Observed means are based on *n* = 3 replicates for multi-species products and *n* = 4 for single-species products. ^3^ One-sample *t*-test comparing each observed mean with the declared log_10_ value (α = 0.05). ^4^ Acceptance threshold defined as ±0.5 log_10_ CFU, following Italian Ministry of Health guidance. ^5^ Relative difference = ((observed − declared)/declared) × 100%. ^6^ Declared counts marked “min.” represent manufacturer minimums; positive deviations above these values are compliant.

### 2.4. Sample Preparation, Culturing and Enumeration

For each probiotic, three independent aliquots (multi-species products) or four independent aliquots (single-species products) were weighed and processed in parallel. One gram of each poultry product, or one human capsule/tablet (contents emptied if encapsulated), was suspended in 20 mL sterile phosphate-buffered saline (PBS, pH 7.4; Sigma, P4417) containing three 6 mm sterile metal beads and vortex-mixed for 2–3 min until fully dispersed. Ten-fold serial dilutions (up to 10^−9^) were prepared in the same PBS; 20 µL of each dilution was spread onto the appropriate selective-agar sector (Table 1) with sterile L-shaped spreaders (Thermo Scientific, Loughborough, UK, P12322048). Cultured dilutions of *Bacillus subtilis* products underwent an additional 80 °C for 10 min heat activation step before plating, using a heat block (TECHNE, model DB100/3). Plates were incubated under genus-specific conditions (17–72 h, temperature and atmosphere per Table 1). Anaerobic plates were placed in 2.5 L Oxoid AnaeroJar™ jars (Thermo Scientific, Basingstoke, UK) with AnaeroGen™ sachets and resazurin indicator strips to verify anaerobiosis. Colony counts were expressed as CFU per gram, capsule or tablet using the recorded colony number and dilution factor. Negative controls—blank agar plus PBS only, plated in duplicate—were included in every run to verify the sterility of media, diluent and equipment.

### 2.5. Species Identification

Bacterial identification followed a three-step workflow: (i) growth on the selective agars listed in Table 1; (ii) presumptive recognition by colony morphology (shape, colour, size); and (iii) confirmation by matrix-assisted laser desorption/ionisation time-of-flight mass spectrometry (MALDI-TOF MS).

After enumeration, ≥4 colonies were picked from each selective agar on at least two replicate plates for each probiotic product. Fresh colonies were transported aseptically on the same day to Freeman Hospital, Newcastle upon Tyne, UK, and analysed on a Bruker Biotyper instrument under NHS laboratory protocols. For each isolate, a well-isolated colony was transferred to a 96-spot stainless-steel target plate (Bruker), overlaid with 1 µL α-cyano-4-hydroxycinnamic acid (HCCA) matrix solution (Bruker), and allowed to air-dry. Spectra were acquired within 10 min using the manufacturer’s default settings and matched against the Bruker reference library.

Identification scores were interpreted with Bruker cut-offs: >2.0 = species level; 1.70–1.99 = secure genus level; <1.70 = unreliable [21].

### 2.6. Statistical Analysis

Observed colony-forming-unit (CFU) counts (per g, capsule or tablet) were first summarised in their original scale as means ± standard deviation (SD). Descriptive statistics are presented in Table 3. Counts were then converted to log_10_ CFU per unit. Prior to hypothesis testing, the normality of each log_10_-transformed dataset was assessed by the Ryan–Joiner test, with no dataset showing a significant departure from normality (*p* > 0.05). One-sample *t*-tests on the log_10_-transformed means assessed whether each product differed from its declared log_10_ label value, using α = 0.05 for significance. For comparisons with label claims, *t*-based 95% confidence intervals (CIs) are reported in Table 4 alongside *p*-values.

In line with the Italian Ministry of Health guidance on probiotics and prebiotics [8,22], a label claim was deemed acceptable if the observed log_10_ count remained within ±0.5 log_10_ CFU, a margin intended to cover viable count loss over shelf life. Although the UK has no formal tolerance for probiotic viability, the Italian Ministry of Health guideline is the only European document that specifies a quantitative limit (±0.5 log_10_ CFU) for shelf-life losses. We therefore adopted this well-recognized benchmark as a pragmatic reference for products sold on the UK market. All analyses were performed in Minitab software v21.4.

## 3. Results

Poultry probiotics generally met or exceeded label claims (Table 2). For example, P5 (*Bacillus subtilis*) delivered 1.40 × 10^10^ CFU g^−1^ versus the declared 2 × 10^9^ CFU g^−1^, and P11 likewise exceeded its minimum declaration. In contrast, four of five human products under-delivered the declared CFU count (Table 3). Most notably, P1 yielded no colony growth on any agar plate, despite testing composite samples equivalent to 1 g (seven tablets), which were labelled as containing 2 × 10^11^ CFU g^−1^. The best-performing human product, P6, slightly exceeded its declaration (3.32 × 10^9^ ± 0.11 CFU capsule^−1^ vs. 3 × 10^9^ CFU capsule^−1^).

**Figure 1:**
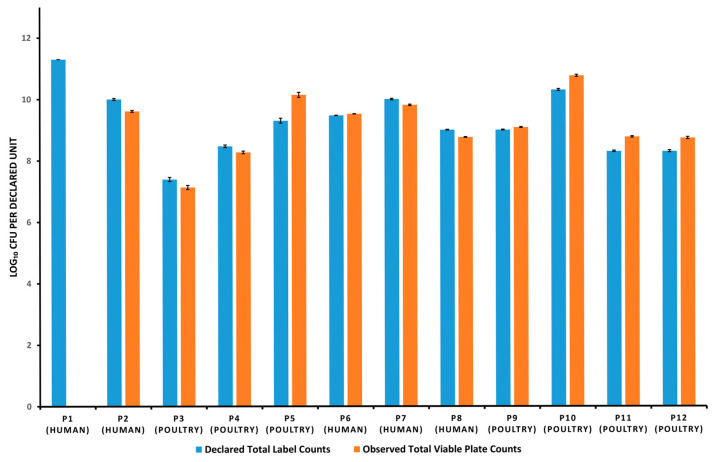
Visual representation of declared versus observed total viable bacterial counts (log_10_ CFU per declared label unit) in Human and Poultry Probiotics (P1–P12). “Declared unit” refers to CFU (colony forming unit) per gram for poultry probiotics, or CFU per capsule/tablet for human products. For P1, both a single tablet and a composite of seven tablets (1 g) were tested, with no growth detected.

For inferential comparisons with label claims, the results are summarized in Table 4, which reports *t*-based 95% confidence intervals (CIs) alongside *p*-values. Relative differences calculated as ((observed − declared)/declared) × 100%—ranged from −3.52% to +9.03% in poultry products and from −100% to +0.53% in human products (Table 4). One-sample *t*-tests on log-transformed counts confirmed significant deviations (*p* < 0.05) for every product; however, all but P1 met the adopted ±0.5 log_10_ benchmark—P5 exceeded the upper margin but was acceptable because its label specifies a minimum viable count (Table 4). A graphical summary of declared versus observed total counts is shown in Figure 1.

MALDI-TOF MS confirmed the labelled species in 10 of 12 products (Table 3 and Table 4). *Bifidobacterium bifidum* was not detected in P2, and Bacillus isolates from P3, P4, P5 and P11 were assigned to the *B. velezensis*/*B. amyloliquefaciens* group—both members of the *B. subtilis* group. No isolates outside the label-declared species were detected by MALDI-TOF MS, indicating that no contamination was present in any of the studied probiotics.

Products P9 and P12 employ ratio-based labelling (3:1:6 for *B. animalis*, *L. salivarius* and *E. faecium*, respectively). Observed counts closely matched these declared ratios, indicating accurate representation of relative composition.

## 4. Discussion

This study evaluated the viability and label accuracy of seven poultry and five human probiotic products by viable plate count, with bacterial identification confirmed by selective agars and MALDI-TOF MS. The primary goal was to verify whether manufacturers’ claims on viable counts and species composition were met or not. Determining effective dose and health impact is product and indication specific and requires targeted in vivo evaluation; these endpoints were not addressed in the present study, and accordingly we do not claim or refute efficacy for any product.

Significant discrepancies were observed between label claims and measured counts, particularly among the human products, in agreement with previous surveys of commercial probiotics [5,6,7,8,10]. For instance, product P1, labelled to contain 2 × 10^11^ CFU g^−1^ of *Lactobacillus acidophilus*, yielded no detectable viable bacteria (Table 3). By contrast, P6 and P7 displayed counts close to their declared values, suggesting that manufacturing practices and strain robustness strongly influence final viability of bacterial presence [23,24].

In contrast to the human products, poultry products generally showed higher accuracy in terms of the declared bacterial types and numbers. This observation likely reflects their formulation with resilient strains, principally *Bacillus* spp. spores, known to tolerate heat, desiccation and gastric acidity [25]. Even the three poultry products lacking *Bacillus* (P9, P10, P12) performed well; all were produced by the same manufacturer and consisted of microencapsulated powders, a technology shown to enhance survival during processing and storage of similar probiotics [23,26]. Among the component species, *Enterococcus faecium* consistently exhibited the greatest viability, out-competing *Lactobacillus salivarius* and *Bifidobacterium animalis* on selective agar. Growth of this robust strain on BSM plates had to be suppressed with lithium-mupirocin, echoing earlier reports [19].

These findings contradict the initial hypothesis, drawn from the literature for human probiotics, that most products would fall short of their label claims. While little contemporary information exists for animal probiotics, earlier North American surveys reported poor compliance for equine [2] and companion animal products [13]. The higher conformity observed here for poultry products may reflect stricter EU regulations on feed additives [27], under which UK poultry probiotics are authorised, versus the weaker dietary-supplement framework governing many human products.

Beyond viability counts, species identification was generally concordant with labels, although *Bifidobacterium bifidum* (P2) and *Lactobacillus bulgaricus* (P6) were not recovered as CFU on agar plates. Absence on selective agar does not prove absence in the product: a low starting abundance or competitive overgrowth by faster-growing strains could mask the recovery of slow-growing strains [28,29]. Nevertheless, for consumers and for regulators, the practical issue is whether the claimed viable dose of each named species is delivered at the use-by date.

The practical implications of this study are significant. For consumers and clinicians, our findings highlight the importance of brand reputation and transparency, suggesting that products providing third-party quality verification may be more reliable. For veterinarians and poultry producers, the data provide confidence in the quality of currently regulated products but also emphasize the need for continued vigilance. For regulatory bodies, this research underscores the potential public health gap between the tightly controlled animal feed sector and the more loosely regulated human supplement market, supporting calls for harmonized standards and more robust post-market surveillance.

However, the findings should be interpreted with some limitations in mind. Because products were chosen by convenience sampling to span common applications and formulations, the panel is not probabilistic; therefore, results should be interpreted as a market snapshot rather than an exhaustive, brand-representative survey. Furthermore, a key limitation is the single-batch sampling per product, which precludes assessment of lot-to-lot variability; future surveillance should ideally include multiple batches to assess manufacturing consistency.

Looking ahead, several avenues for future research are apparent. To build on this market snapshot, longitudinal studies analysing multiple batches of the same product over time are essential to differentiate between sporadic quality control failures and systemic manufacturing issues. Additionally, the integration of molecular methods, such as quantitative PCR (qPCR), alongside plate counting would provide a more complete picture by enabling the quantification of viable but non-cultivable (VBNC) cells. Finally, stability trials that assess viability under simulated consumer storage conditions would offer valuable insights into the real-world performance of these products throughout their shelf life.

Based on these findings, the data highlight the importance of selecting hardy strains and appropriate formulation technologies. For poultry, spore-forming *Bacillus* and microencapsulated blends offer clear viability advantages under farm-level temperature and humidity fluctuations. Human products appear more vulnerable to viability loss, likely due to the use of non-spore-forming lactic acid bacteria that are less resistant to heat, desiccation and oxygen exposure during manufacturing and storage, and may require similar technological upgrades, such as inclusion of spore forming Bacillus strains and advanced microencapsulation techniques, to achieve the reliability seen in the animal sector.

Ultimately, these findings reinforce the necessity of rigorous quality control and standardized testing procedures across probiotic production. Ensuring accurate and transparent labelling of viable counts and species composition is crucial, not only to maintain regulatory compliance but also to uphold consumer trust and maximize the therapeutic potential of probiotic products.

## 5. Conclusions

The present study underlines marked variability in the viability of commercial probiotics sold in the UK, with human formulations displaying the greatest divergence from label claims. Several human products delivered markedly fewer viable bacteria than those declared on the labels, whereas poultry products generally conformed to their labels. This likely reflected the use of spore-forming or microencapsulated strains that better withstand the processing and storage of these products. These findings support calls for stronger regulatory oversight and lot-specific quality control testing to ensure that marketed probiotic products provide the promised health benefits. Future work should focus on refining enumeration methods and transferring proven veterinary technologies, such as microencapsulation and robust *Bacillus* strains, into human probiotics. By aligning manufacturing practices with rigorous viability standards, both the human and animal sectors can deliver more reliable, evidence-based probiotic interventions.

## Data Availability

The data presented in this study are available upon request from the corresponding author. The data are not publicly available due to institutional and ethical restrictions.

## Supplementary Materials

The following supporting information can be downloaded at: https://www.mdpi.com/article/10.3390/microorganisms13081933/s1, Table S1: Storage metadata for probiotic products (P1–P12), including expiry date, label storage instruction, actual storage temperature and storage time prior to testing.
