# Correction: Taha et al. Assessing Bacterial Viability and Label Accuracy in Human and Poultry Probiotics Sold in the United Kingdom. *Microorganisms* 2025, *13*, 1933

**DOI:** 10.3390/microorganisms14030607

**Published:** 2026-03-09

**Authors:** Mostafa Waleed Taha, Danielle J. C. Fenwick, Emma C. L. Marrs, Abdul Shakoor Chaudhry

**Affiliations:** 1 School of Natural and Environmental Sciences, Newcastle University, Newcastle upon Tyne NE1 7RU, UK; m.w.t.taha2@newcastle.ac.uk; 2 Microbiology Research Department, Freeman Hospital, Newcastle upon Tyne NE7 7DN, UK; danielle.fenwick@nhs.net (D.J.C.F.); e.marrs@nhs.net (E.C.L.M.)

In the original publication [1], there was a mistake in the column “Observed Viable Plate Counts (Mean ± SD)” of Table 2. Details and viable counts of commercial poultry probiotic products and Table 3. Details and viable counts of commercial human probiotic products as published. The SD values were presented in a truncated format and should appear as full SD values. The corrected tables appear below.

**Table 2:** Details and viable counts of commercial poultry probiotic products.

Product Code	Species Declared on the Label	Declared Label Total (CFU/g)	MALDI-TOF MS ID ^2^	Observed Viable Plate Counts (Mean ± SD) ^3^
P3	*Bacillus subtilis*	2.5 × 10^7^	*Bacillus* spp.	1.375 × 10^7^ ± 2.06 × 10^6^
P4	*Bacillus subtilis*	3 × 10^8^	*Bacillus* spp.	1.900 × 10^8^ ± 1.82 × 10^7^
P5	*Bacillus subtilis*	2 × 10^9^	*Bacillus* spp.	1.400 × 10^10^ ± 2.58 × 10^9^
P9	*Bifidobacterium animalis*	Ratio ^1^ 3/10	*Bifidobacterium animalis*	1.300 × 10^8^ ± 2.58 × 10^7^
	*Lactobacillus salivarius*	Ratio 1/10	*Ligilactobacillus salivarius*	3.200 × 10^7^ ± 3.65 × 10^6^
	*Enterococcus faecium*	Ratio 6/10	*Enterococcus faecium*	1.055 × 10^9^ ± 4.43 × 10^7^
	Total bacteria	1 × 10^9^	Total bacteria	1.212 × 10^9^ ± 4.47 × 10^7^
P10	*Enterococcus faecium*	2 × 10^10^	*Enterococcus faecium*	5.800 × 10^10^ ± 4.54 × 10^9^
P11	*Bacillus subtilis*	2 × 10^8^	*Bacillus* spp.	5.950 × 10^8^ ± 3.10 × 10^7^
P12	*Bifidobacterium animalis*	Ratio ^1^ 3/10	*Bifidobacterium animalis*	1.080 × 10^7^ ± 1.48 × 10^6^
	*Lactobacillus salivarius*	Ratio 1/10	*Ligilactobacillus salivarius*	1.375 × 10^6^ ± 2.21 × 10^5^
	*Enterococcus faecium*	Ratio 6/10	*Enterococcus faecium*	5.300 × 10^8^ ± 4.32 × 10^7^
	Total bacteria	2 × 10^8^	Total bacteria	5.418 × 10^8^ ± 4.22 × 10^7^

^1^ For products P9 and P12, the manufacturer declared the total CFU per gram of product but did not specify individual CFU counts for each bacterial species. Instead, the label listed each species as a ratio of the total bacterial population. ^2^ Identification of isolates by matrix-assisted laser desorption/ionisation time-of-flight mass spectrometry. ^3^ Mean ± standard deviation per gram. Observed means are based on *n* = 3 replicates for multi-species products and *n* = 4 for single-species products.

**Table 3:** Details and viable counts of commercial human probiotic products.

Product Code	Form	Species Declared on the Label	Declared Label Total (CFU/Form ^1^)	MALDI-TOF MS ID ^3^	Observed Viable Plate Counts (Mean ± SD) ^4^
P1	Tablets	*Lactobacillus acidophilus*	2 × 10^11^	NA	No CFU detected ^2^
P2	Capsule	*Lactobacillus acidophilus*	ND ^5^	*Lactobacillus acidophilus*	3.483 × 10^9^ ± 2.76 × 10^8^
		*Bifidobacterium animalis*	ND	*Bifidobacterium animalis*	6.533 × 10^8^ ± 3.51 × 10^7^
		*Bifidobacterium bifidum*	ND		
		Total bacteria	1 × 10^10^	Total bacteria	4.137 × 10^9^ ± 2.96 × 10^8^
P6	Capsules	*Lactobacillus acidophilus*	ND	*Lactobacillus acidophilus*	3.300 × 10^9^ ± 1.00 × 10^8^
		*Lactobacillus salivarius*	ND	*Lactobacillus salivarius*	2.067 × 10^6^ ± 4.61 × 10^5^
		*Bifidobacterium animalis*	ND	*Bifidobacterium animalis*	2.100 × 10^7^ ± 2.00 × 10^6^
		*Lactobacillus Bulgaricus*	ND		
		Total bacteria	3 × 10^9^	Total bacteria	3.320 × 10^9^ ± 1.01 × 10^8^
P7	Capsules	*Lactobacillus acidophilus*	ND	*Lactobacillus acidophilus*	5.667 × 10^9^ ± 3.05 × 10^8^
		*Bifidobacterium bifidum*	ND	*Bifidobacterium bifidum*	8.100 × 10^8^ ± 4.35 × 10^7^
		Total bacteria	1 × 10^10^	Total bacteria	6.477 × 10^9^ ± 3.36 × 10^8^
P8	Chewable Tablets	*Lactobacillus acidophilus*	ND	*Lactobacillus acidophilus*	5.533 × 10^8^ ± 1.52 × 10^7^
		*Bifidobacterium animalis*	ND	*Bifidobacterium animalis*	2.800 × 10^7^ ± 1.00 × 10^6^
		Total bacteria	1 × 10^9^	Total bacteria	5.813 × 10^8^ ± 1.56 × 10^7^

^1^ Per form (capsule or tablet, as specified in the “Form” column, except for P1, where label showed counts per gram). ^2^ For product P1, viability testing was conducted using two sample types: (i) a single tablet, and (ii) a composite of seven tablets equivalent to 1 g, as the label declared viable count per gram. No colony growth was observed in either case on any agar medium. ^3^ Identification of isolates by matrix-assisted laser desorption/ionization time-of-flight mass spectrometry. ^4^ Mean ± standard deviation per form. Observed means are based on *n* = 3 replicates for multi-species products and *n* = 4 for single-species products. ^5^ ND = Not declared on label; the manufacturer provided a total count but did not specify counts for individual species.

The authors state that the scientific conclusions are unaffected. This correction was approved by the Academic Editor. The original publication has also been updated.
